# Darwinizing Gaia: conceptual approaches

**DOI:** 10.1098/rstb.2024.0089

**Published:** 2025-08-07

**Authors:** W. Ford Doolittle

**Affiliations:** ^1^ Department of Biochemistry and Molecular Biology, Dalhousie University, Halifax, Nova Scotia, Canada B3H 4R2

**Keywords:** Gaia hypothesis, natural selection, multispecies community

## Abstract

After briefly describing James Lovelock’s Gaia hypothesis, I will argue that Gaia does not reproduce, or rather that it has what Peter Godfrey-Smith would call ‘too many parents’ to undergo natural selection according to Lewontin’s Recipe. So the hypothesis did not and still does not make sense to most Darwinians. If (i)Lewontin’s Recipe were extended to include differential persistence as well as differential reproduction, (ii) part/whole relationships were accepted as a form of ‘reproduction’, or (iii) the ‘gene’s-eye view’ of Richard Dawkins as further extended by David Hull and us were adopted, then the Gaia hypothesis, and lesser claims about some multispecies communities, holobionts and ecosystems, *would* make sense. This last is what the *It’s the song not the singer(s*) theory aimed to do.

This article is part of the discussion meeting issue ‘Chance and purpose in the evolution of biospheres’.

## 1. Introduction

This is meant to be a summary of a book, *Darwinizing Gaia: natural selection and multispecies community evolution* [1]. This essay is a shorter and updated version of that book, with different emphases. As my thinking has become simpler, the case for multispecies community selection, even of Gaia, has become more compelling for me, at least insofar as making it consistent with Darwinian formulations. I am of course aware that Tim Lenton and colleagues have long been pursuing mathematical models for Gaian behaviour [2–4], but this essay (and my book) are purely verbal endeavours. Still, some of these mathematical treatments may embody elements of what is presented here.

I include among these Darwinian formulations Richard Dawkins’ *The selfish gene* and his *The extended phenotype*, though these books are currently often dismissed as being too reductionist and ‘agential’ about genes [5,6]. I think, however, that evolution by natural selection (ENS) permits of several formulations [7]. Not all of these actually satisfy Darwin’s apparent initial goal, that being to explain *complex* adaptedness (of the vertebrate eye, for instance) in terms only of natural laws. One formulation does, though, and that’s Dawkins’ and Hull’s gene’s-eye view, as articulated in [8] .

## 2. What Lovelock proposed and why most Darwinians thought it was nonsense

James Lovelock proposed that the Earth, in both its biotic and abiotic parts, exhibits homeostatic feedback loops. This implied (through its use of evolved ‘homeostases’), that Gaia was the product of some kind of ENS. Sometimes Lovelock even ventured that Gaia is an ‘organism’, though he, not a Darwinian, did not specify how ENS was involved in its creation.

Dawkins published *The selfish gene* at about the same time (1976 versus 1979 for Lovelock’s first book for the general public, *Gaia: a new look at life on Earth* [9]). But most Darwinians were already under the influence of George Williams’ 1966 opus [10], which emphasized that selection on genes was always to be preferred as explanation. Dawkins doubled-down on this; so Darwinians were almost all opposed to Gaia.

Genes of course ‘replicate’, while sexual organisms only ‘reproduce’, but both show ENS. So in fact Darwinians (most of them) were willing to compromise between Williams’ and Dawkins’ reductionist gene’s-eye view and the more traditionally Darwinian organism-centred understanding (which many still simultaneously held) by adopting Lewontin’s Recipe [11], which is as follows (in its 1970 version):

— Different individuals in a population have different morphologies, physiologies and behaviours (phenotypic variation).
— Different phenotypes have different rates of survival and reproduction in different environments (differential fitness).
— There is a correlation between parents and offspring in the contribution of each to future generations (fitness is heritable).

There is thus a clear dependence on lineages—that parents, be they genes of guinea pigs or the guinea pigs themselves, produce offspring, and they in turn produce grand-offspring, and so forth. Both genic replication and what sexual organisms do —reproduce (with offspring more-or-less resembling one or the other or both parents)— are encompassed in Lewontin’s formulation. But note, ENS *does* depend on there being lineages in the first place.

Lineage dependence is also exhibited by multilevel selection theory (MLST), in which reproduction is an essential component of ENS. In fact, in my view MLST is just an entailment of Lewontin’s Recipe. Peter Godfrey-Smith [12, p. 2161] could thus say (in 2012):

Evolution by natural selection is change in a *population* owing to variation, heredity and differential reproductive success. … the criteria required are abstract; genes, cells, social groups and species can all, in principle, enter into change of this kind. For any objects to be *units of selection* in this sense, however, they must be connected by parent–offspring relations; they must have the *capacity to reproduce*.

So *non-reproduction* was the trouble with the Gaia hypothesis, as Lovelock formulated it. For Lovelock, Gaia, or at least its biotic part, was to be *a unit of selection*, but it could not be that because it does not leave lineages and (which is the nearly same thing) does not reproduce. Its parts (species or organisms within them) of course do, but as a collective it does not, and so it cannot as a collective be a unit of selection, and cannot have been selected for homeostatic adaptations as a collective. Collectives do not form lineages, at least of any simple kind (see below). The same trouble was recently got into by holobiont enthusiasts: and in a sense they have the same fight with Darwinians, all over again.

Later, Godfrey-Smith added a qualification to Lewontin’s Recipe [12, p. 2161], and this will be important to my argument:

… there must be some parent-offspring similarity, and the clarity of a ‘parentoffspring’ relation of the relevant kind is inversely related to the number of parents—if there are too many parents there are no parents at all.

## 3. But do not ecosystems actually reproduce, anyway?

Elliott Sober, in a now deleted critique of a paper I wrote with Chris Lean and Joe Bielawski [13], wrote that ‘in fact, a daughter community of size *n* is descended from up to *n* parent communities. This resembles biparental inheritance in the case of sexual organisms.’ Asexual organisms of course have only one parent defining their lineages but sexual species do have two: the egg donor and the sperm donor. So ecosystems, specifically those that are assembled de novo each time, might differ from sexual species only in having still more ‘parents’, namely the several ecosystems that gave rise to the individual organisms recruited by the next generation of ‘offspring’ ecosystems, these individual organisms playing the role of gametes. This is the nature of the resemblance to biparental inheritance.

The problem, as Godfrey-Smith prefigured in his later caveat, is that the number of parents in a multispecies collective such as a recurring ecosystem can easily get to be ‘too many’. For sexual species, this problem is usually solved by having only two parents per offspring. The genes that make an offspring green or wrinkly (two separate traits, by the way) come most of the time from a single egg-donor and a single sperm-donor, one or both of which the offspring inevitably resembles more than it does most unrelated individuals. Interestingly, these genes also necessarily come (if unlinked) from any two of four grandparents, any two of eight great-grandparents and so forth. Green is eventually unlinked to wrinkly. So there get to be ‘too many’ donors of alleles, eventually. But there are not too many parents in the first generation for any trait: offspring do preferentially resemble their parents over all. The ultimate mixing is thanks to inter-chromosomal and intra-chromosomal recombination, which many people think is the real reason for sex.

But for multispecies communities, ecosystems and holobionts showing horizontal inheritance, which many of the latter do, this mixing happens in the very first generation. Each parent of the many individuals recruited to form ‘offspring’ generations of communities can be different. So there can easily get to be ‘too many’. That is why multispecies communities are not thought to be subject to ENS: there is no particular parent or limited number of parents that an offspring resembles, so Lewontin’s Recipe does not work, or works only on a trait-by-trait basis and assumes something like Gause’s Law (no two species with exactly the same trait). Perhaps that this happens too after several generations in a sexual population is why population genetics deals first with single alleles.

## 4. Fixing this

**Figure 1.**
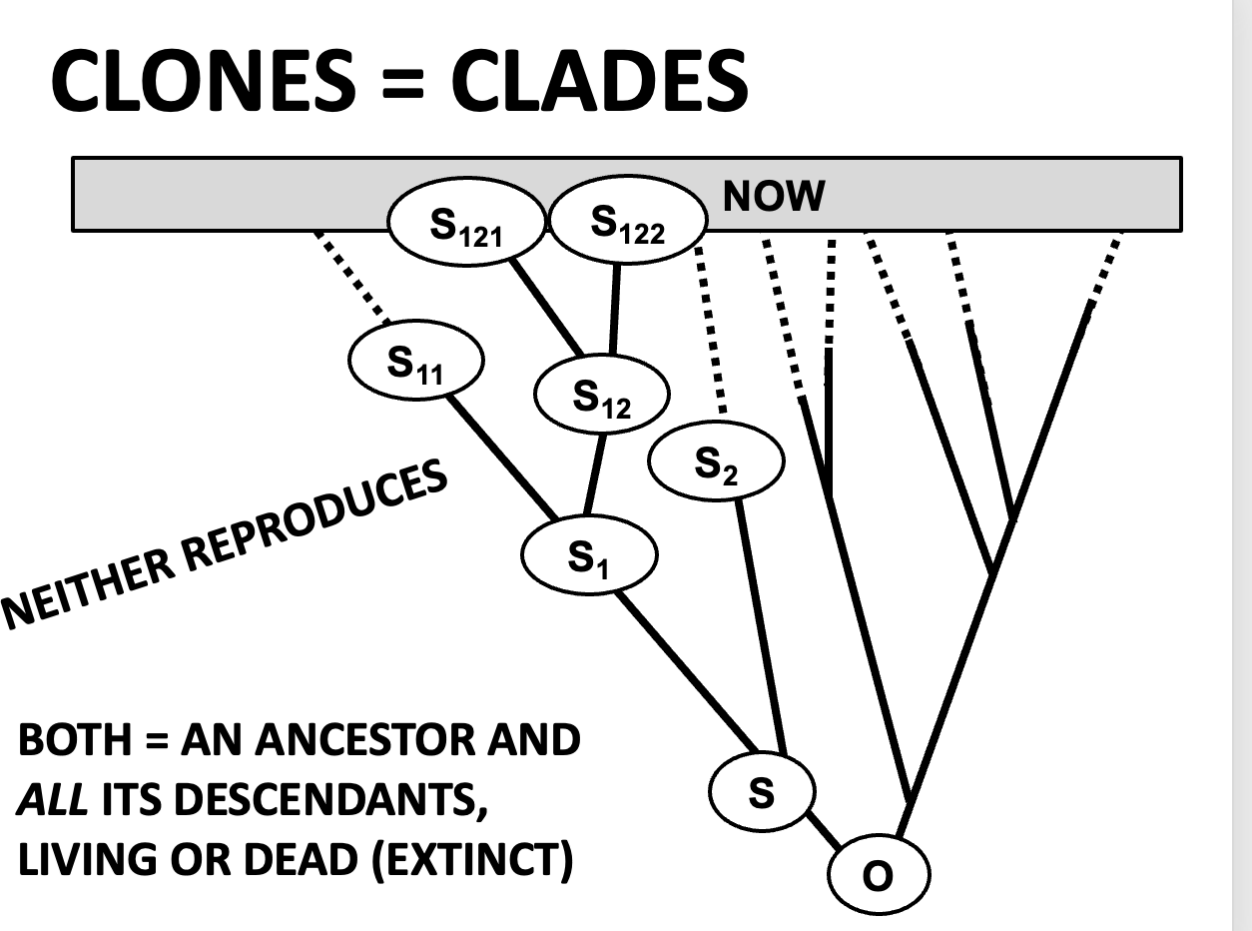
The analogy between clones and clades.

One way to align Lovelock’s Gaia hypothesis with some brands of traditional Darwinian thinking has been the subject of several papers on clade selection. Clade selection is just selection for differential survival (persistence) of clades: clades do not reproduce, by definition. The most recent of these papers [14] is probably the clearest. It analogizes clade survival to clone survival in a chemostat, a device often used to maintain growing cells (of bacteria for instance) at a constant cell density. At any given time, all cells in an idealized chemostat compete, directly or indirectly, to be the ancestor of all cells at some future time (that future being sooner if there is selection). This is just a consequence of coalescent theory. Such competition can be at the cellular level—the cell that replicates itself faster wins, because it uses available substrates more efficiently, for instance (figure 1).

But that cell produces a clone of cells, and sometimes it is properties that can be defined *only* at the clone (or clade) level that are under selection. For instance, one way for a cell to mutate might be to have a variable optimal growth temperature (sometimes different from that of the chemostat). The result would be that the mutation is selected *against* at the cellular (organismal) level when the chemostat is held at a constant temperature: it is bad to be different, and some mutated cells would not replicate at all. But clones that are more variable will outlast others if the temperature of the chemostat varies. This would be as modelled in Pierrick Bourrat’s 2015 paper entitled ‘Levels of selection are artefacts of different fitness temporal measures’ [15]. In other words, if a mutation is disfavoured at the within-species level but is favoured at the between-species (higher, clone or clade) level, then, depending on the strengths of the two selections and the sizes of populations at both levels, it could be favoured overall. This is what I think many of the heated arguments concerning whether or not ‘group selection’ even exists are *actually* about [16]. It is all about whether there can be ‘downward causation’: I think that there can.

Positive selection at any level is *always* reducible to differentially replicating genes, but evolutionary biology (and certainly MLST) seems to need the notion that selection can be implemented as a causal explanation at several levels, even though these levels do in the end show ontological dependence [17] consistent with the reductionism of Williams or Dawkins. So, we must allow what Sober & Wilson [18] call William’s Principle—‘that adaptation at a level requires selection at that level’—but we must also admit that we have a situation in which there is *explanatory autonomy* coupled with *ontological dependence* [14,17] if we are to stick to a reductionist ontology. That is, we must allow for selection to be a causal explanation at multiple levels, even while we admit that genes will always be responsible.

So we could have situations in which mutability is selected a*gainst* at the cellular or species level but *for* at the clone (or clade) level, because it makes the resultant clone (or clade) more variable and thus more likely to survive in competition with other less variable ones in an environmentally fluctuating chemostat (or in nature). Whether or not alleles favouring mutability increase in frequency requires that we look at both level-specific explanations. This of course is what several group-selectionist publications understand to be what sex and recombination are ultimately *for*. Group (between-species) selection dominates, and it can be for persistence or survival, not reproduction.

## 5. A clade-selection (differential-persistence) argument for Gaia

A clone is a single ancestor and all its descendants, as is a clade. Neither can reproduce, so there is no ‘too many parents’ problem. Clones and clades can only persist longer than other clones or clades, or fail to and thus go extinct—which is the death of all cells, or the extinction of all species. Molecular phylogeneticists now assume that the clade consisting of the last universal common ancestor, or LUCA, and its descendants encompasses all recognized Bacteria, Archaea or Eukarya currently on Earth. So all constituents of current or recently extinct earthly life are one of the three, by definition [19].

**Figure 2.**
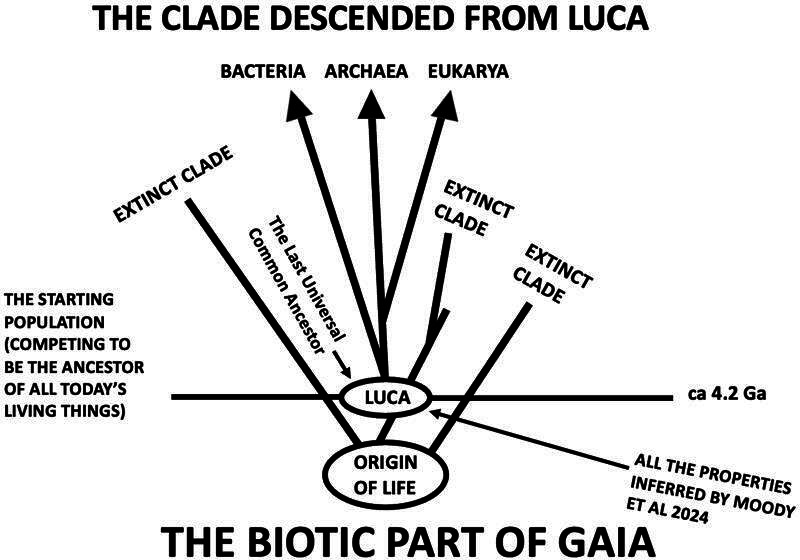
The clade that is now all of life. Moody et al. (2024) refers to [20].

But LUCA was already a fairly sophisticated cell, judging from all the systems and molecules currently present in all Bacteria, Archaea and Eukarya [20]. Most of the components of the translational apparatus were, for instance, already in place, as were many of the protein systems or molecules responsible for energy use and generation. So LUCA must have had competitors, cellular entities, wannabe LUCAs, that somehow failed in their attempt to be the ancestor of all current living creatures. These were part of LUCA’s ‘population’. And of course, if one clone/clade is to out-persist others, there need to have *been* others, at least potentially and initially [1]. This idea is illustrated in figure 2.

Here is where the chemostat model seems most relevant: there is *always* a population of cells competing to be the ancestor of all cells in the future [14]. In a sense, the clone generates it, continuously. Similarly, there are always members of a clade all of whose descendants die: that is the meaning of extinction. The founder of all species in the future (the equivalent of LUCA) is the single member of that population that did not. Although there may not be a competing *reproductive* population at the level at which selection is imposed, at a lower level there *always* is. Moreover, before an ancestor and all its descendants comprises all there is in the chemostat or in nature, it is inevitably part of a dwindling population of competitors (‘sub-clones’ or ‘sub-clades’) for that honour. So there *is* a population of competitors, just as there is for an allele that is ultimately to be fixed in population genetics. Whether or not there is ‘reproduction’ (implicit in Lewontin’s Recipe) depends on whether or not sub-clone or sub-clade propagation counts as a form of it. One sub-clone or sub-clade ultimately becomes the ancestor of all entities in the future, just as LUCA became the ancestor of all things alive today.

Differential survival (of sub-clones or sub-clades) could in principle have been by chance, but if Janković & Ćirković are right (as I think they are) in their paper entitled ‘Evolvability is an evolved ability’, there were *reasons* that LUCA and its descendants survived [21]. Among these could have been a shared biochemistry (making most organisms edible by some other organism) and lateral gene transfer (the ability to benefit from the acquisition of other organisms’ genes, facilitated by a shared genetic code). So, likely, the clade of LUCA *differentially* persisted on the basis of some of its sub-clade-level properties, and the likeliness of *Homo sapiens* extinguishing all other species on Earth—that all of biodiversity is at risk—now seems unreasonable. The species making up the clade of LUCA and its descendants are now ecologically diverse.

Clone or clade-level properties—cell or species richness, ecological diversity, geographical distribution and mutual aid (edibility and gene transfer)—are what clones or clades are selected *for* at this ‘higher’ level, and thus the products of adaptation at that level, according to Williams’s Principle. All are caused by some difference at a lower level, but none is the reason that entities survive at that lower level. So we might add differential persistence to differential reproduction (lineage formation) in Lewontin’s Recipe and recast the resemblance of an entity at one time to itself at another (perdurance of its properties) as a form of inheritance.

After all, ENS is just an increase in the frequency of selected types. It can be achieved by either a relative increase in the number of those types, or a decrease in the number of unselected types. In either case, there is *downward causation* in the sense that there are more species (or cells) with the selected property than there would otherwise have been, because that property was selected *for* at the higher level. Campbell [22, p. 180] defines this force when he writes ‘processes at the lower levels of a hierarchy are restrained by and act in conformity to the laws of the higher level*’.* And of course the clade comprising LUCA and its descendants just *is* Gaia, or the biotic component thereof.

## 6. Differential persistence also works in the replicator/interactor framework of David Hull

Instead of reformulating Lewontin’s Recipe, however, we could adopt David Hull’s replicator/interactor framework [23]. Insofar as this has developed into what is called the ‘gene’s-eye view’, it licenses us to interpret Godfrey-Smith’s ‘too many parent’ caveat as applying only on a trait-by-trait, allele-by-allele (replicator-by-replicator) basis, although leaving the issue of ‘What is a trait?’ unresolved.

**Figure 3.**
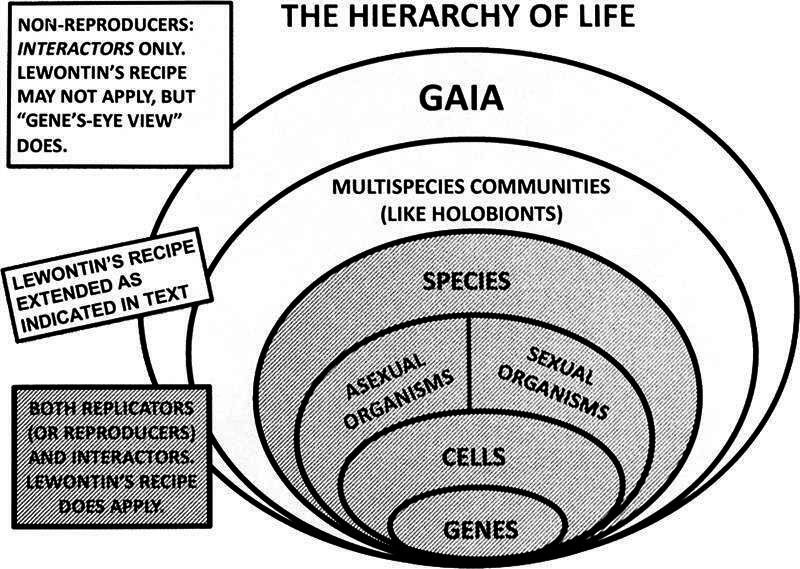
The hierarchy of life, an expanded version of David Hull’s replicator/interactor theory.

As to the replicator/interactor framework more generally, David Hull [23, p. 318] wrote that ENS is just ‘a process in which the differential extinction and proliferation of interactors cause the differential perpetuation of the replicators that produced them’. So the clade that is the biotic part of Gaia could just be the interactor whose ‘differential extinction and proliferation cause the differential perpetuation of the [many] replicators that produce it’ [23, p. 318]. Its interactions with the environment have to do with the number, ecological diversity, geographical distribution and evolvability of its constituent species, all of which serve to perpetuate those ‘replicators’ by ‘downward causation’. How this would work is shown in figure 3.

The replicators in this case would be the species that make up the clade, expanding on the definition given by Hull, who had in mind mostly Dawkinsian genes [24]. But insofar as whole bacterial cells and genomes replicate, the interactor-phenotype-determining genes are not the only ones that benefit (are differentially perpetuated). For sexual species with recombination, however, it may be only the later (and those genes closely linked to genes selected at higher levels) that benefit. There is a gradient of benefit, depending on degrees of epistasis and linkage. It is in fact on this model that more sophisticated forms of modern population genetics are based.

And David Hull’s interactor/replicator framework is what our earlier theoretical intervention in the case of holobionts was based, although we did not realize this at the time. That theory (‘It’s the song, not the singer(s)’, or ITSNTS) is described most fully in Doolittle & Inkpen [25]. It goes like this. Interaction patterns or processes are themselves ‘units of selection’ by virtue of the fact that they ‘recruit’ (encourage the evolution of) genes or the organisms or the species that can perform one or more of their interactions or steps. So non-reproducing interactors can evolve by what looks like natural selection but differently from Lewontin’s Recipe. Okasha says this very well

when he says [26, p. 58] that ‘for MLS1 to produce sustained evolutionary consequences, collectives must ‘reappear’ regularly down the generations, but there is no reason why the collectives themselves must stand in parent–offspring relations.’ Damuth and Heisler [27, p. 407] defined MLS1 and MLS2 as follows: *Of interest in the former case are the effects of group membership on individual fitnesses, and in the latter the tendencies for the groups themselves to go extinct or to found new groups (i.e., group fitnesses).* Okasha had precisely these processes in mind, I think.

But MLS1 just is the replicator/interactor framework, viewed appropriately [1]. Interactors increase in frequency through a process that looks very much like selection by differential reproduction, but is not. Or if it is (as in my third section), it often has ‘too many parents’ [12].

Lateral gene transfer encourages the evolution of interactors (as in figure 3), but simple species diversification is adequate: there are more taxa and relevant genes facilitating the parts of an interaction or the steps in a process than there would have otherwise been because of the persistence (re-production, not necessarily reproduction) of that overall pattern or process. Properties of this pattern/process promote its ability to recruit taxa to perform it, and these properties are under selection, for persistence. So the pattern/process is the interactor, while the genes or taxa having them are the replicators. There may not now be a ‘population’ of interactors, but the replicators that compete to form them clearly do make up populations, as in our clone/clade analogy.

This may be what Hull meant. Interactors with selected-for properties do increase in frequency, because they ‘cause the differential perpetuation of the replicators that produce’ them. Interactors are, in a sense, the ‘manifestors of adaptation’ [7]. Thus the frequency of recruitment of these replicators into the next generation of interactors increases, even if randomly. And often there will be selection for higher frequency (higher than random) at the level of replicators, which *are* subject to Lewontin’s Recipe [28].

## 7. Darwinizing Gaia: conclusion

If we want to ‘Darwinize’ Lovelock’s Gaia hypothesis, we cannot use an unmodified form of Lewontin’s Recipe, because Gaia, if it is to be seen as ‘reproductive’, has what Godfrey-Smith calls ‘too many parents’. The same is true for many multispecies communities. Lewontin’s Recipe and its extension as MLST do cash out ‘fitness’ as differential reproduction. We can either modify that recipe, allowing differential persistence to join differential reproduction as a process or outcome of ENS and substituting identity over time for ‘heritability’, or embrace the ‘gene’s-eye view’, which focuses on genes and the traits they are responsible for. Both are compatible with the earlier theory called ‘It’s the song, not the singer(s)’, and requires that we view Gaia and other multispecies communities as interacting with their environments because of level-specific properties.

## Data Availability

This article has no additional data.
